# Whole genome sequencing of 35 individuals provides insights into the genetic architecture of Korean population

**DOI:** 10.1186/1471-2105-15-S11-S6

**Published:** 2014-10-21

**Authors:** Wenqian Zhang, Joe Meehan, Zhenqiang Su, Hui Wen Ng, Mao Shu, Heng Luo, Weigong Ge, Roger Perkins, Weida Tong, Huixiao Hong

**Affiliations:** 1 Division of Bioinformatics and Biostatistics, National Center for Toxicological Research, U.S. Food and Drug Administration, 3900 NCTR Road, Jefferson, AR 72079, USA; 2 University of Arkansas at Little Rock/University of Arkansas for Medical Sciences Bioinformatics Graduate Program, Little Rock, Arkansas, AR 72204, USA

**Keywords:** genetics, sequencing, genome, variant, population, Korean

## Abstract

**Background:**

Due to a significant decline in the costs associated with next-generation sequencing, it has become possible to decipher the genetic architecture of a population by sequencing a large number of individuals to a deep coverage. The Korean Personal Genomes Project (KPGP) recently sequenced 35 Korean genomes at high coverage using the Illumina Hiseq platform and made the deep sequencing data publicly available, providing the scientific community opportunities to decipher the genetic architecture of the Korean population.

**Methods:**

In this study, we used two single nucleotide variant (SNV) calling pipelines: mapping the raw reads obtained from whole genome sequencing of 35 Korean individuals in KPGP using BWA and SOAP2 followed by SNV calling using SAMtools and SOAPsnp, respectively. The consensus SNVs obtained from the two SNV pipelines were used to represent the SNVs of the Korean population. We compared these SNVs to those from 17 other populations provided by the HapMap consortium and the 1000 Genomes Project (1KGP) and identified SNVs that were only present in the Korean population. We studied the mutation spectrum and analyzed the genes of non-synonymous SNVs only detected in the Korean population.

**Results:**

We detected a total of 8,555,726 SNVs in the 35 Korean individuals and identified 1,213,613 SNVs detected in at least one Korean individual (SNV-1) and 12,640 in all of 35 Korean individuals (SNV-35) but not in 17 other populations. In contrast with the SNVs common to other populations in HapMap and 1KGP, the Korean only SNVs had high percentages of non-silent variants, emphasizing the unique roles of these Korean only SNVs in the Korean population. Specifically, we identified 8,361 non-synonymous Korean only SNVs, of which 58 SNVs existed in all 35 Korean individuals. The 5,754 genes of non-synonymous Korean only SNVs were highly enriched in some metabolic pathways. We found adhesion is the top disease term associated with SNV-1 and Nelson syndrome is the only disease term associated with SNV-35. We found that a significant number of Korean only SNVs are in genes that are associated with the drug term of adenosine.

**Conclusion:**

We identified the SNVs that were found in the Korean population but not seen in other populations, and explored the corresponding genes and pathways as well as the associated disease terms and drug terms. The results expand our knowledge of the genetic architecture of the Korean population, which will benefit the implementation of personalized medicine for the Korean population.

## Introduction

Genetics is the key to deciphering the phenotypic diversity of different populations. Given that it is impossible for two individuals to have exactly identical genomes, even a monozygotic twin pair, decoding sequence information becomes the first and most important step in searching for genetic factors leading to phenotypic diversity. Since the 3 billion base pairs of the human genome were published in 2003 [1,2], many types of genetic studies have been conducted [3,4].

Taking advantage of microarrays, genome-wide association studies (GWAS) have been utilized to identify common variants associated with different phenotypes among populations [5-7]. Next generation sequencing (NGS) enables us to identify not only common variants but also rare variants at a relatively low cost [8-15]. Many studies have already used whole genome sequencing to explore the whole spectrum of the genomic variation in both healthy individuals [16,17] and individuals associated with clinical indications [18-20], aiming at uncovering the complexities of the genome and possible clinical associations. The HapMap Project [21] and the 1000 Genomes Project (1KGP) [22] are two large efforts at deciphering the variants in various populations by microarray and NGS, respectively. More than 38 million variants were identified from 1,887 normal samples of 17 populations by HapMap [23,24] and 1KGP [25,26], providing comprehensive references of variants for genetic studies.

The Korean population is an Asian population not included in HapMap or 1KGP. The first study decoding the Korean genome was published in 2009 [27]. Thereafter, several publications further revealed the genetic variation within the Korean population [28,29]. GeVab [30] (http://gevab.org/) is a genome browser integrating all the variants (including 3.44 million SNPs) identified from the first Korean genome. However, to our knowledge, no comprehensive genetic architecture for the Korean population has been constructed to decipher the unique Korean population genetic features.

Here, we aimed at utilizing whole genome sequencing data to characterize the genetic features of the Korean population. Through comparative analyses with genetic variants detected by HapMap and 1KGP, we identified the SNVs only contained in the Korean population and explored their associations with functional pathways, disease terms and drug terms. The findings deepen our understanding of the genetics and evolution of the Korean population and are expected to facilitate personalized medicine for the Korean population.

## Materials and methods

### Study design and workflow

**Figure 1:**
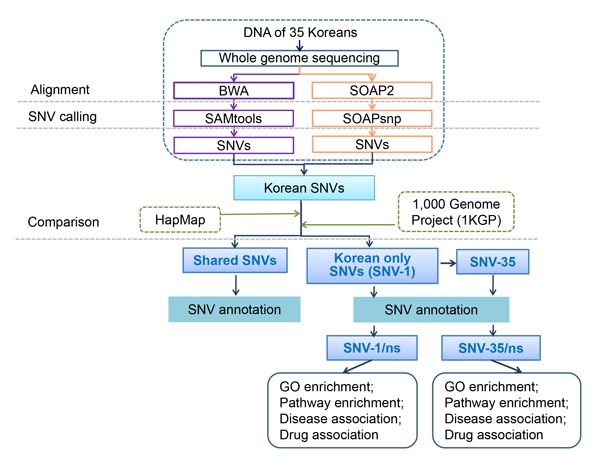
**Study design and workflow of this study**. The whole genome sequencing data of 35 individuals were used for mapping to the human genome and SNV calling by two pipelines. The overlapped SNVs from the two pipelines were used to represent Korean population and then compared with two references to search for Korean only SNVs and shared SNVs with other populations. Then shared SNVs were annotated. For the Korean only SNVs, two subgroups (SNV-1 and SNV-35) were derived in accordance with the occurrences in the Korean population. The two subgroups of SNVs were then annotated. The non-synonymous SNVs were determined and the corresponding genes were used for enrichment and association analyses.

The raw reads of whole genome sequencing of 35 Korean individuals were analyzed by two SNV calling pipelines. One used BWA [31] for mapping reads and SAMtools [32] for SNV calling; the other used SOAP2 [33] for mapping reads and SOAPsnp [34] for SNV calling. For each of the two pipelines, SNVs for each of the 35 Korean individuals were identified. The SNVs from the 35 individuals were merged together according to their genomic positions separately for the two pipelines. The SNVs were then compared between pipelines. Only those SNVs detected by both pipelines in the 35 individuals were considered to have relatively high quality and termed as Korean SNVs for the subsequent analyses. The remaining SNVs were detected by only one pipeline and deemed to be of low quality and not used in subsequent analysis. Next, we compared the Korean SNVs with the SNVs detected from other populations in 1KGP and HapMap and divided the Korean SNVs into two types: the SNVs detected in at least one of the 35 Korean individuals but not identified in other populations in HapMap and 1KGP (termed as SNV-1) and the SNVs detected in both Korean population and other populations (termed as shared SNVs). Both shared SNVs and Korean only SNVs were then annotated separately. According to the annotations, non-synonymous SNVs in Korean only SNVs were determined and then the involved genes were identified to perform gene ontology and KEGG pathway enrichment analyses and to explore their associations with diseases and drugs. The workflow is shown in Figure 1.

### Source of data

The whole genome sequencing data from the Korean Personal Genome Project (KPGP) (http://opengenome.net/index.php/Main_Page) are publicly available and can be downloaded from http://opengenome.net/index.php/Korean. We obtained the data via the Critical Assessment of Massive Data Analysis (CAMDA) consortium (http://dokuwiki.bioinf.jku.at/doku.php/start). The data contained whole genome sequencing raw reads, BWA alignment results, and SAMtools SNV-calling results. The raw reads were generated from an Illumina Hiseq system using paired-end sequencing with 90 base pairs (bp) read length. There were 38 samples, which included one Caucasian female and her two children. The remaining 35 samples were of Korean descent and used in this study. In addition, we downloaded the SNVs (in hg19 format) of nine Korean individuals from TIARA (http://tiara.gmi.ac.kr/download) that were detected from whole genome sequencing data [28].

### Alignment

Two sets of alignment results were generated for the raw reads of the 35 Korean samples. The first set was provided by KPGP and was generated by using BWA (version 0.5.9) to map raw reads to the human genome (hg19) with 45bp seed sequence allowed (see Additional file 1 for details). The second set of alignment results was generated in our laboratory, using SOAPaligner/SOAP2 (version 2.21) and mapped the raw reads to the human genome reference (ftp://hgdownload.cse.ucsc.edu/goldenPath/hg19/bigZips/chromFaMasked.tar.gz) (hg19) with no indels and at most five mismatches allowed (see Additional file 1 for details).

### SNV calling

Two different SNV calling pipelines were run against the two sets of alignment results to call SNVs in each individual. The first set of SNV calling results were generated by KPGP based on their alignment results from BWA. The SNVs were called using mpileup command of SAMtools (version 0.1.16) with depth ≥5 (termed as SAMtools SNVs for simplicity) (see Additional file 1 for details). The second SNV calling pipeline using SOAPsnp (version 1.05) was applied by our laboratory. SNVs from this pipeline were termed as SOAPsnp SNVs (see Additional file 1 for details). In this pipeline, duplicated reads were removed from the raw reads alignment of SOAP2 and the resultant aligned reads were used for SNV calling by SOAPsnp with options: -r 0.0005 -e 0.001 -t -u -L 90 -Q i. Next, filters of quality score (≥20), neighbor distance (≥5) and depth (≥3) were applied to the SNV calling results.

### Comparing SNVs between two pipelines

The SNVs of all 35 samples were combined to generate a merged SNVs file for each pipeline. If a SNV was not detected in a sample, then we assigned the reference allele to that sample at that location. The merged SNV files were compared between pipelines according to genomic positions of SNVs. The SNVs with overlapped genomic positions between the two files were deemed to be of relatively high confidence and were named as Korean SNVs for subsequent analyses. The genotypes for each Korean SNV were determined based on the results from SOAPsnp.

### Preprocessing raw genotype files from HapMap and 1KGP

The genotypes of 1,092 individuals in 1KGP were downloaded from ftp://ftp.1000genomes.ebi.ac.uk/vol1/ftp/phase1/analysis_results/integrated_call_sets/. SNVs were extracted from the raw genotype files with the requirement of "VT=SNP" in the "INFO" column for 23 chromosomes (autosome chromosomes and X chromosome) and no requirement for Y chromosome. The Perl program is included in Additional file 1.

The genotypes of 1,417 individuals from HapMap were downloaded from ftp://ftp.ncbi.nlm.nih.gov/hapmap/genotypes/2010-08_phaseII+III/ (Feb 12, 2013). First, raw genotype files from different populations were merged together to generate a consensus genotype file. The 424 locations that were indels or had non-unique rsIDs were removed from the consensus genotype file. Since HapMap provided SNP positions based on human genome hg18 version, while SNV positions of 1KGP and our results were based on hg19 version, we converted the positions of HapMap SNVs from hg18 version to hg19 version using liftOver [35]. The SNVs that failed to be converted were then compared with 1KGP. If a SNV had the same rsID in 1KGP and HapMap, then the hg19 position of this SNV in 1KGP was assigned to the SNV in HapMap. The reference allele of each SNV was determined by searching the sequence of hg18 human genome (downloaded from ftp://ftp.ncbi.nih.gov/genomes/H_sapiens/ARCHIVE/BUILD.36.3/Assembled_chromosomes/, on March 22, 2013).

### Determining shared SNVs and Korean only SNVs

Among Korean SNVs, 1KGP and HapMap, 134 SNVs were found to have at least two rsIDs. We required that one SNV have only one rsID, and therefore randomly selected one rsID for such SNVs and discarded the remaining lines of such SNVs from the three genotype files.

Next, we compared the SNVs from the three datasets according to their hg19 positions. If the same SNV was included in both Korean SNVs and 1KGP (or in both Korean SNVs and HapMap), then the SNV was categorized as a shared SNV, otherwise the SNV was categorized as a Korean only SNV.

### Determination of occurrences in population of SNVs

The occurrence of each SNV in a population was defined as the number of samples that contained the SNV in the population. For the Korean only SNVs, we used SNV-i to present the set of SNVs with occurrences ≥ i.

### SNP annotation

We used VEP [36] (release 75) to annotate the SNVs. The file "homo_sapiens_vep_75.tar.gz" (ftp://ftp.ensembl.org/pub/release-75/variation/VEP/homo_sapiens_vep_75.tar.gz) was used as the reference file for annotation. The detailed source list for the reference file can be found at http://useast.ensembl.org/info/genome/variation/sources_documentation.html#homo_sapiens. Specifically, in this version of the reference file, the gene symbols originated from RFAM, Uniprot_gn, miRBase, HGNC, Clone_based_vega_gene, and Clone_based_ensembl_gene. The non-synonymous SNVs were defined as the SNVs with labels of "missense_variant", "stop_gained", "stop_lost", "stop_retained_variant", "coding_sequence_variant", "initiator_codon_variant", "incomplete_terminal_codon_variant", "splice_donor_variant", "splice_acceptor_variant" and "splice_region_variant" in the "Consequence" column of VEP annotation results. We thereafter named the non-synonymous SNVs in SNV-i as SNV-i/ns.

### Calculating SNV count per gene

For each of the four SNV sets (SNV-1, SNV-35, SNV-1/ns, and SNV-35/ns), all of the involved genes were used to study the number of identified SNVs found in each gene. We then ranked the genes according to their SNV counts.

### Functional enrichment analyses

Gene ontology and KEGG pathway enrichment analyses were conducted for genes of SNV-i/ns using WebGestalt [37,38]. Associations of the SNV-i/ns involved genes with disease terms and drug terms were identified using WebGestalt, which were actually based on the predictions from GLAD4U (http://bioinfo.vanderbilt.edu/glad4u/). The GLAD4U algorithm first queries all the disease (drug) terms from PharmGKB in the MEDLINE database to retrieve the corresponding publications, next determines the gene-to-publication link according to Entrez Gene information, and finally returns the genes significantly associated with the query disease (drug) terms. The associated references of WebGestalt included Gene Ontology (version 1.2, 11/11/2012), KEGG (03/21/2011), PharmGKB (1/26/2013) and GLAD4U (1/26/2013). The hypergeometric test was used for enrichment evaluation analysis. Multiple test adjustment was performed using the BH (Benjamini & Hochberg) method [39] (https://stat.ethz.ch/R-manual/R-devel/library/stats/html/p.adjust.html).

## Results and discussions

### Read alignment and SNV calling

A total of 3.8 TB raw whole genome sequencing data from 35 Korean samples was acquired from KPGP, with depth in each sample ranging from 30X to 42X (mean = 36X) (Additional file 2). Using the aligner BWA, KPGP mapped 93.41% of raw reads to the human reference genome (Additional file 2). Based on the alignment results, KPGP then identified an average of 3,421,868 SNVs in each individual using SAMtools (Additional file 2). Merging all the identified SNVs in each individual yielded a final set of 9,119,633 SAMtools SNVs.

In our pipeline, 91.01% of raw reads were mapped to the human reference genome by SOAP2 (Additional file 2). Using the mapped reads, SOAPsnp then identified an average of 3,395,688 SNVs in each individual (Additional file 2). By merging all the SNVs identified in each individual, a total of 9,964,511 SOAPsnp SNVs were identified.

### Determining Korean SNVs

Given the still difficult task of detecting accurate SNVs with a single calling tool, and that SNVs detected by multiple pipelines will likely have relatively higher accuracy [40], we used the overlapped set of SNVs from the two pipelines to represent the Korean population. After comparing SOAPsnp SNVs with SAMtools SNVs in terms of genomic positions, we identified a total of 8,555,726 Korean SNVs, covering 85.86% of SOAPsnp SNVs and 93.82% of SAMtools SNVs. The SNV density was almost uniform in each chromosome except sex chromosomes (Additional file 3).

### Comparing SNVs from different populations

We then compared the Korean SNVs with two public references, 1KGP and HapMap, to search for Korean only SNVs. We found that 2,677,812 (31.3%) Korean SNVs were covered by both 1KGP and HapMap (Additional file 3); 10,791 (0.13%) Korean SNVs overlapped HapMap SNV calls only, while 4,653,510 (54.39%) Korean SNVs overlapped 1KGP only (Additional file 3). Finally, we identified 1,213,613 Korean only SNVs (SNV-1) after the comparison, representing 12.18% of the Korean SNVs. From the SNV-1, 12,640 SNVs with occurrences = 35 (SNV-35) were identified (Additional file 4).

### Korean SNVs and Korean only SNVs frequency features

**Figure 2:**
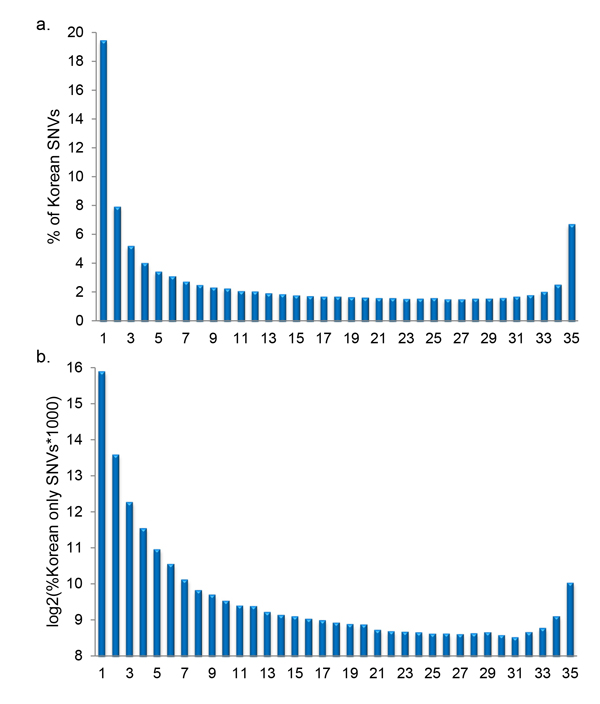
**Frequency distribution of Korean SNVs and Korean only SNVs**. Frequency distributions in terms of occurrences in the population were calculated and plotted for Korean SNVs (a) and Korean only SNVs (b). The x-axis indicates the occurrences and the y-axis gives the corresponding SNV frequency in %.

We computed the occurrence rate of Korean SNVs and Korean only SNVs among the 35 individuals with results plotted in Figures 2a and 2b, respectively. The plots show the percent of SNVs versus the number of the 35 individuals sharing the SNVs. Figure 2a shows that nearly 20 % of Korean SNVs are observed in only one individual. We deemed the singular SNVs occurrences to be of lower confidence and reliability, as they are most likely to be attributable to errors in sequencing, reference genome or analyses. Similarly, confidence and reliability of SNVs likely increases as prevalence of an individual SNV increases in the 35 individuals. Nearly 7% (571,571) of SNVs occur in all 35 individuals. Between 1.5% and somewhat more than 2% of SNVs are prevalent in 10 to 34 individuals. Figure 2b shows that nearly 60.59 % (735,271) of Korean only SNVs are observed in only one individual, while 1.04% (12,640) of Korean only SNVs are observed in all 35 individuals. Therefore, we rationally conjectured that Korean only SNVs prevalent across many of the individuals would be more important for treating Koreans differently from other populations in the personalized medicine.

### SNV frequency in different populations

For each of the 7,331,322 SNVs detected in both the Korean population and 1KGP, we calculated its frequency for the Korean population and for each of the 14 populations included in 1KGP. The frequency distributions of the 15 populations are plotted in Additional file 5. For SNVs with frequency larger than 0.2, the distributions are very similar, while the distributions of SNVs having frequency less than 0.2 are relatively different. The results suggested that low frequency SNVs can better characterize populations than high frequency SNVs.

### Assessing reproducibility of Korean only SNVs

Ju *et al*. [28] conducted whole genome sequencing of 10 Korean individuals at 26.1-fold coverage and identified 3.45 to 3.73 million SNVs from each individual by aligning reads to the human genome reference hg18. We downloaded the SNVs from 9 individuals that were converted to hg19 by the authors and used those SNVs to assess reproducibility of the Korean only SNVs detected in this study. We detected 3,318,098 to 3,444,114 (SOAPsnp) and 3,369,094 to 3,480,518 (SAMtools) SNVs from each of the 35 Koreans (Additional file 2), similar to Ju's result. We further assessed reproducibility of the Korean only SNVs by identifying the Korean only SNVs contained in the SNVs from Ju *et al *[28]. Additional file 4 lists the reproducibility values for SNV-i, revealing that the SNVs detected in more samples are more reproducible.

### Substitution mutational spectrum in the Korean population

**Figure 3:**
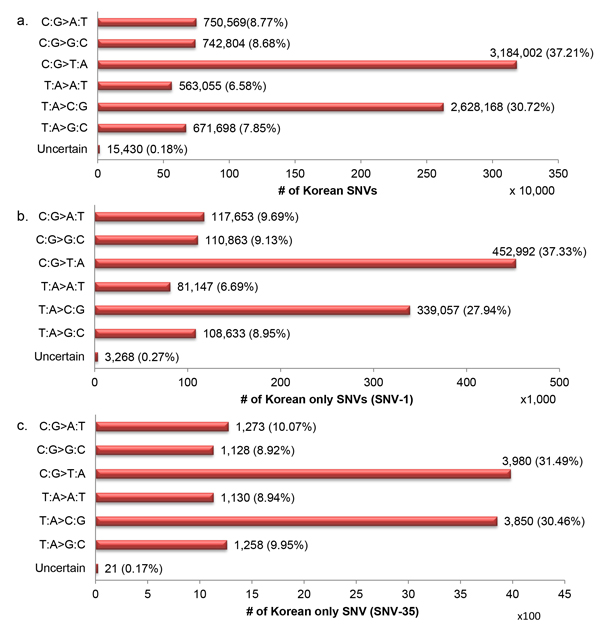
**Mutation spectrum of Korean SNVs**. The base substitution types of SNVs in the: (a) Korean SNVs, (b) Korean only SNVs (SNV-1) and (c) Korean only SNVs (SNV-35) are on the y-axis, and SNV number for category is on x-axis. Percentages in the parentheses give proportion among all SNVs. SNVs with multiple alternative alleles were considered to be SNVs with uncertain mutation type.

We evaluated the mutation spectrum on both the Korean SNVs and Korean only SNVs (SNV-1 and SNV-35). For Korean SNVs, the transition/transversion ratio was 2.1 (Additional file 6), the same as the expected ratio for the human genome calculated using whole genome sequencing data [41]. According to the SNV compositions (Figure 3), we found that the most prevalent changes in all three SNV sets was C:G->T:A transition, the same trend as the raw base substitution mutational spectrum in humans [42]. In addition, we found that Korean SNVs and SNV-1 occurred more frequently at C/G base pairs, which was consistent with previous findings [42]. Nevertheless, almost half of SNV-35 mutations occurred at T/A base pairs.

### SNVs with potential functional consequences

**Figure 4:**
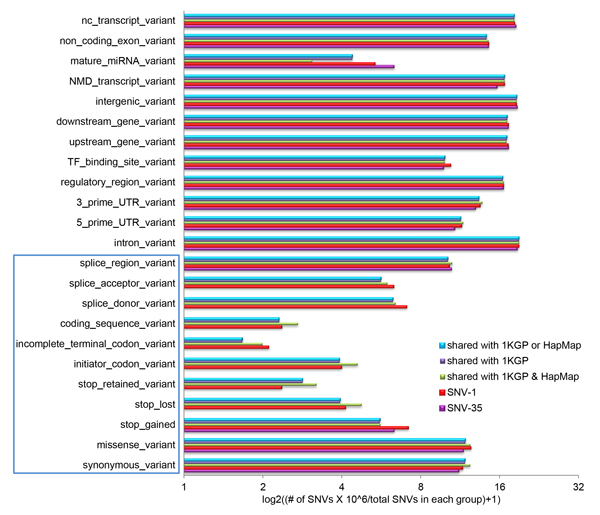
**Annotation of Korean SNVs**. Annotation using VEP [36], of different group: SNVs-1 (the red bars), SNV-35 (the purple bars), shared SNVs between Korean SNVs and 1KGP or between Korean SNVs and HapMap (the cyan bars), shared SNVs between Korean SNVs and 1KGP (the dark purple bars), shared SNV between Korean SNVs, 1KGP and HapMap (the dark yellow bars). The x-axis gives number of SNVs in log2 transformation and y-axis gives the annotation terms.

One of the major objectives of this study was finding Korean only SNVs with potential functional consequences that differentiated the Korean population from other populations. To this end, we annotated all the Korean SNVs by separating them into different groups, including (1) Korean only SNVs (SNV-1), (2) Korean only SNVs (SNV-35), (3) shared SNVs between Korean SNVs and 1KGP, (4) shared SNVs among Korean SNVs, 1KGP and HapMap, and (5) shared SNVs between Korean SNVs and 1KGP or between Korean SNVs and HapMap. We found that the majority (89.46%) of Korean SNVs came from introns followed by intergenic regions (Figure 4 andTable 1). The total SNVs located in these two regions were 87.79% for SNV-1, 83.35% for SNV-35, 90.02% for SNVs among Korean SNVs, 1KGP and HapMap, 89.74% for SNVs shared between Korean SNVs and 1KGP, and 89.73% for SNVs shared between Korean SNVs and 1KGP or between Korean SNVs and HapMap. In addition, we found noncoding transcript SNVs to be third most prevalent.

**Table 1:** Annotations of Korean only SNVs and shared SNVs

Category		SNV-1^1 ^(%)	SNV-35^2 ^(%)	Shared with 1KGP & HapMap (%)	Shared with 1KGP (%)	Shared with 1KGP or HapMap (%)
CDS^3 ^and splicing regions	Synonymous Variant	3,587 (0.296)	29 (0.229)	13,978 (0.522)	25,988 (0.354)	26,030 (0.355)
	Missense Variant	6,620 (0.545)	40 (0.316)	14,147 (0.528)	26,835 (0.366)	26,909 (0.367)
	Stop Gained Variant	173 (0.014)	1 (0.008)	129 (0.005)	343 (0.005)	348 (0.005)
	Stop Lost Variant	20 (0.002)	0 (0)	70 (0.003)	105 (0.001)	106 (0.001)
	Stop Retained Variant	5 (0.000)	0 (0)	22 (0.001)	45 (0.001)	45 (0.001)
	Initiator Codon Variant	18 (0.001)	0 (0)	62 (0.002)	104 (0.001)	104 (0.001)
	Incomplete Terminal Codon Variant	4 (0.000)	0 (0)	8 (0.000)	16 (0.000)	16 (0.000)
	Coding Sequence Variant	5 (0.000)	0 (0)	15 (0.001)	29 (0.000)	29 (0.000)
	Splice Donor Variant	162 (0.013)	0 (0)	226 (0.008)	557 (0.008)	562 (0.008)
	Splice Acceptor Variant	95 (0.008)	0 (0)	165 (0.006)	357 (0.005)	359 (0.005)
	Splice Region Variant	1,496 (0.123)	18 (0.142)	3,983 (0.149)	8,298 (0.113)	8,312 (0.113)

Regulatory region and adjacent regions to CDS	5' Prime UTR^4 ^Variant	3,358 (0.277)	22 (0.174)	8,462 (0.316)	19,526 (0.266)	19,568 (0.267)
	3' Prime UTR Variant	13,638 (1.124)	99 (0.783)	37,145 (1.387)	76,397 (1.042)	76,547 (1.043)
	Regulatory Region Variant	117,783 (9.705)	1,186 (9.383)	267,822 (10.002)	649,464 (8.859)	650,799 (8.864)
	TF Binding Site Variant	1,645 (0.136)	11 (0.087)	2,511 (0.094)	6,913 (0.094)	6,927 (0.094)
	Upstream Gene Variant	185,499 (15.285)	2,010 (15.902)	343,491 (12.827)	989,954 (13.503)	991,984 (13.511)
	Downstream Gene Variant	192,188 (15.836)	1,999 (15.815)	370,173 (13.824)	1,028,139 (14.024)	1,030,243 (14.032)

non-coding regioins	NMD^5 ^Transcript Variant	127,138 (10.476)	634 (5.016)	287,027 (10.719)	779,516 (10.633)	780,572 (10.631)
	Mature miRNA Variant	48 (0.004)	1 (0.008)	20 (0.001)	146 (0.002)	146 (0.002)
	Noncoding Exon Variant	28,539 (2.352)	292 (2.310)	62,513 (2.334)	143,214 (1.953)	143,617 (1.956)
	Noncoding Transcript Variant	376,646 (31.035)	4,590 (36.313)	815,372 (30.449)	2,191,354 (29.890)	2,195,528 (29.903)

Intron Variant		602,464 (49.642)	5,272 (41.709)	1,379,647 (51.521)	3,703,303 (50.513)	3,708,842 (50.515)

Intergenic Variant		462,916 (38.144)	5,264 (41.646)	1,030,973 (38.501)	2,876,064 (39.230)	2,879,598 (39.220)

Total SNVs		1,213,613	12,640	2,677,812	7,331,322	7,342,113

^1^SNV-1: SNVs with occurrences ≥ 1 in all of 35 samples.

^2^SNV-35: SNVs with occurrences = 35 in all of 35 samples.

^3^CDS: coding DNA sequence

^4^UTR: untranslated region

^5^NMD: Nonsense Mediated decay

Although the majority of the SNVs belonged to the top three ranked categories, we still found many SNVs that would alter the amino acid code (Table 1): 12,185 in SNV-1, 88 in SNV-35, 32,805 in shared SNVs among Korean SNVs, 1KGP and HapMap, 62,677 in shared SNVs between Korean SNVs and 1KGP, and 62,820 in shared SNVs between Korean SNVs and 1KGP or between Korean SNVs and HapMap. Interestingly, of these, 70.56%, 67.04%, 57.39%, 58.54%, 58.56% were non-synonymous SNVs, respectively in the categories, which would change the amino acid code and therefore alter the protein (Table 1). These results indicated that, although SNVs shared by Korean, 1KGP and HapMap have similar distributions of SNV categories within CDS and splicing regions, the Korean only SNVs (SNV-1 and SNV-35) have higher percentages of total non-synonymous SNVs than shared SNVs in these two regions; this implied a potential importance of these non-synonymous SNVs in differentiating Korean from other populations.

### Korean only SNVs gene enrichment

**Figure 5:**
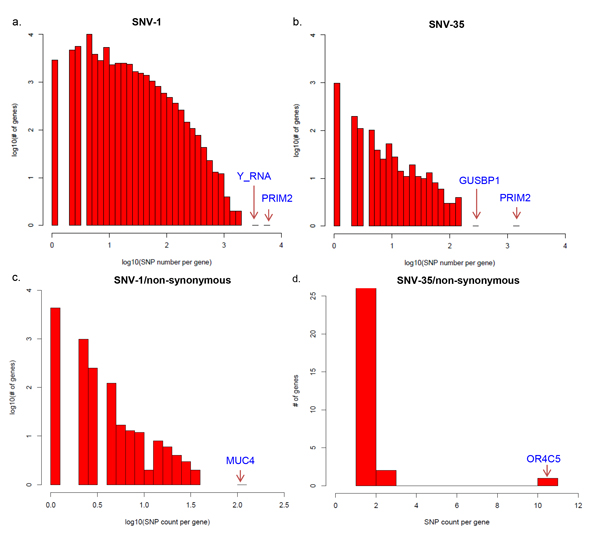
**Distribution of count of Korean SNVs per gene**. The count of SNVs per gene was calculated for SNVs in SNV-1 (a), SNV-35 (b), SNV-1/ns (c) and SNV-35/ns (d). Top one or two ranked genes were labeled. The x-axis indicates SNVs per gene in log10 transformation and the y-axis depicts number of genes.

We studied the number of SNVs per gene for Korean only SNVs. A total of 53,771 genes were covered by 1,213,613 Korean only SNVs (SNV-1), some of which had an especially high number of Korean only SNVs, e.g. PRIM2 (5,033 SNVs) (Figure 5a,Additional file 7). When considering the 12,640 SNVs of SNV-35, we also found a total of 1,640 genes involved and PRIM2 had an extremely high number of SNVs (1,470 SNVs) (Figure 5b andAdditional file 7). In addition, we evaluated the number of SNVs per gene in the non-synonymous SNVs (8,361 for SNV-1/ns and 58 for SNV-35/ns). Some 5,754 (10.7% of 53,771) genes were found to be involved in SNV-1/ns and 37 (2.3% of 1,640) in SNV-35/ns (Additional file 7). As shown in Figures 5c and 5d, MUC4 (116) and OR4C5 (11) had enriched non-synonymous SNVs from SNV-1 and SNV-35, respectively.

### Gene ontology analysis of non-synonymous Korean only SNVs

We clustered genes associated with non-synonymous SNVs in SNV-1 and SNV-35 in order to search for GO term enrichment. We found the most enriched GO terms were motor activity (p-value with multiple test adjustment (adjP) = 6.40e-12) for non-synonymous SNV-1 and system process (adjP= 0.0150) for non-synonymous SNV-35 (Additional file 8).

### Non-synonymous Korean only SNVs pathway analysis

We analyzed pathway enrichment using genes associated with non-synonymous SNVs in SNV-1 and SNV-35. We found metabolic pathway (adjP = 2.61e-37) was the top enriched pathway in non-synonymous SNVs in SNV-1, followed by ECM-receptor interaction (adjP = 7.90e-20) (Additional file 9). For SNV-35, we found that requiring at least two genes be observed in the pathway resulted in only three pathways hits, vibrio cholera infection (adjP = 0.0018), axon guidance (adjP = 0.0051) and olfactory transduction (adjP = 0.0279) (Additional file 9).

### Non-synonymous Korean only SNVs disease association analyses

We analyzed disease association by WebGestalt using the genes involved in non-synonymous SNVs in SNV-1 and SNV-35. Briefly, we searched the genes for associations with disease terms of PharmGKB. We found adhesion (adjP = 3.44e-59), disease susceptibility (adjP = 1.21e-36) and genetic predisposition to disease (adjP = 5.23e-35) were the most prevalent disease terms associated with SNV-1/ns (Table 2 and Additional file 10). Nelson syndrome (adjP = 0.0102) was associated with SNV-35/ns. Therefore, we inferred that special attention should be made for the Korean population when treated for the above mentioned terms related diseases, since other populations did not carry those Korean only SNVs. To assess robustness of the significantly associated disease terms identified, we conducted the same association analysis for SNV-i, i = 2 to 10. The top 10 significantly associated disease terms as well as their corresponding multiple text adjusted p-values are listed in Additional file 11. Close analysis of the significantly associated disease terms revealed that the terms are stable with an average 55% of terms sharing by two SNV-i (i = 2 to 10) and the larger the i value, and the smaller the difference between two i values, the more terms are shared (Additional file 12).

**Table 2:** Top associated disease terms with non-synonymous SNVs in SNV-1 and SNV-35.

Korean only SNVs	Order	Disease term	Gene count	%of all genes in the category	P value (raw)	P value (adjusted)
SNV-1/ns	1	Adhesion	241	37.249	2.28E-62	3.44E-59
	2	Disease Susceptibility	238	28.848	1.60E-39	1.21E-36
	3	Genetic Predisposition to Disease	231	28.589	1.04E-37	5.23E-35
	4	Myocardial Infarction	90	37.190	4.95E-24	1.87E-21
	5	Urologic Diseases	98	34.386	4.48E-23	1.13E-20
	6	Subarachnoid Hemorrhage	54	51.923	4.30E-23	1.13E-20
	7	Metabolic Diseases	162	26.471	6.55E-23	1.41E-20
	8	Kidney Diseases	95	34.672	1.04E-22	1.74E-20
	9	Skin and Connective Tissue Diseases	137	28.482	1.04E-22	1.74E-20
	10	Nervous System Diseases	176	25.360	1.48E-22	2.23E-20

SNV-35/ns	1	Nelson syndrome	3	0.446	0.0102	0.0102

### Non-synonymous Korean only SNVs drug association analyses

Using WebGestalt, we identified associations with drugs for the genes involved in non-synonymous SNVs in SNV-1 and SNV-35. Similar to the disease association analysis, we searched the drug terms of PharmGKB for associations. Results showed adenosine (adjP = 1.24e-18) to be the drug term most associated with SNV-1/ns, followed by adenosine triphosphate (adjP = 2.61e-13), and immune globulin (adjP = 2.88e-13) (Table 3 and Additional file 11). We found mennitol (adjP = 0.0001), niflumic acid (adjP = 0.0001) and adenosine monophosphate (adjP = 0.0022) are the most associated drug terms for SNV-35/ns (Table 3 and Additional file 13). Based on our analysis, we hypothesize that Koreans might have different responses to the above mentioned terms-related drugs, when compared to other populations. More follow-up studies are required to confirm our findings.

**Table 3:** Top associated drug terms with non-synonymous SNVs in SNV-1 and SNV-35.

Korean only SNVs	Order	Drug term	Gene count	%of all genes in the category	P value (raw)	P value (adjusted)
SNV-1/ns	1	adenosine	133	27.883	3.47E-21	1.24E-18
	2	adenosine triphosphate	87	29.097	1.46E-15	2.61E-13
	3	immune globulin	145	23.237	2.42E-15	2.88E-13
	4	hydroxyurea	30	46.154	9.01E-12	8.04E-10
	5	glutathione	84	24.633	7.75E-11	4.61E-09
	6	phosphoric acid	50	31.447	6.76E-11	4.61E-09
	7	heparin	55	29.255	1.72E-10	8.77E-09
	8	bupropion	38	35.185	3.41E-10	1.52E-08
	9	rosuvastatin	43	32.090	6.95E-10	2.76E-08
	10	calcium chloride	27	40.909	2.70E-09	9.64E-08

SNV-35/ns	1	mannitol	2	10	8.23E-05	0.0001
	2	niflumic acid	2	10	8.23E-05	0.0001
	3	adenosine monophosphate	2	1.961	0.0022	0.0022

## Conclusions

This study characterized the SNVs of 35 Korean individuals through comparing them with SNVs from 17 other populations. Many Asian populations have been sequenced in past years, contributing ever more to deciphering the specific features of variants in each population. Our work adds more valuable insights toward a more thorough characterization of Korean variants. We identified a total of 1,213,613 Korean only SNVs, 12,640 of which occurred in all of the 35 samples. The mutation spectrum of Korean SNVs was in accordance with expectations [42]. Some 10.58% of Korean SNVs were located in exonic regions (Table 1). SNV-1 had 8,361 non-synonymous variants and their involved genes were found enriched in some metabolic pathways. SNV-35 contained 58 non-synonymous variants. Enrichment analysis also found adhesion to be the disease term most associated with SNV-1, while Nelson syndrome was the only disease term associated with the SNV-35. Also, we found that Korean only SNVs were in genes most associated with the drug term adenosine. Altogether, this study should expand our knowledge of the genetic variants in the Korean population, contributing to the development of personalized medicine for this population.

## List of abbreviations used

KPGP: Korean Personal Genomes Project; 1KGP: 1000 Genome Project; SNV: single nucleotide variant; SNV-1: SNVs detected in at least one of the 35 Korean individuals but not included in either HapMap or 1KGP; SNV-35: SNVs detected in all of 35 Korean individuals but not included in either HapMap or 1KGP; SNV-1/ns: non-synonymous SNV from SNV-1; SNV-35/ns: non-synonymous SNV from SNV-35; GWAS: genome-wide association studies; NGS: next generation sequencing; CAMDA: the Critical Assessment of Massive Data Analysis; CDS: Coding DNA Sequence; UTR: untranslated region; NMD: nonsense mediated decay.

## Competing interests

The authors declare that they have no competing interests.

## Authors' contributions

WZ performed all calculations and data analysis, and wrote the first draft of manuscript. JM, ZS, HN, HL, MS, and WG contributed to the data analysis, verified the calculations. RP, WT and HH wrote the final manuscript. HH developed the original idea and guided the data analysis and presentation of results. All authors read and approved the final manuscript.

## Disclosure

The findings and conclusions in this article have not been formally disseminated by the US Food and Drug Administration (FDA) and should not be construed to represent the FDA determination or policy.

## Supplementary Material

Additional file 1 **Supplementary Methods**. Click here for file

Additional file 2 **Supplementary Table S1 Basic statistics of raw reads and results of read mapping and SNV calling using two pipelines**. Click here for file

Additional file 3 **Supplementary Table S2 Number of Korean SNVs and Korean only SNVs in each category**. Click here for file

Additional file 4 **Supplementary Table S3 A list of SNV number per chromosome as well as SNV density per chromosome**. Click here for file

Additional file 5 **Supplementary Figure S1 SNV frequency distributions in 15 populations**. Frequency distributions in terms of occurrences in the populations were calculated for the SNVs detected in both Korean and the 14 populations from 1KGP. Populations: KOR (35 Koreans in our study), ASW (people with African ancestry in Southwest United States), CEU (Utah residents with ancestry from Northern and Western Europe), CHB (Han Chinese in Beijing, China), CHS (Han Chinese South, China), CLM (Colombians in Medellin, Colombia), FIN (Finnish in Finland), GBR (British from England and Scotland, UK), IBS (Iberian populations in Spain), JPT (Japanese in Tokyo, Japan), LWK (Luhya in Webuye, Kenya), MXL (people with Mexican ancestry in Los Angeles, California), PUR (Puerto Ricans in Puerto Rico), TSI (Toscani in Italia), and YRI (Yoruba in Ibadan, Nigeria). Click here for file

Additional file 6 **Supplementary Table S4 A list of numbers of transition SNVs and transversion SNVs per sample**. Click here for file

Additional file 7 **Supplementary Table S5 A list of genes involved in Korean SNVs (SNV-1, SNV-35, SNV-1/ns and SNV-35/ns) and the SNV count per gene**. Click here for file

Additional file 8 **Supplementary Table S6 Detailed information of top GO terms enriched in non-synonymous Korean only SNVs (SNV-1/ns and SNV-35/ns)**. Click here for file

Additional file 9 **Supplementary Table S7 Detailed information of top pathways enriched in non-synonymous Korean only SNVs (SNV-1/ns and SNV-35/ns)**. Click here for file

Additional file 10 **Supplementary Table S8 Detailed information of top disease terms associated with non-synonymous Korean only SNVs (SNV-1/ns and SNV-35/ns)**. Click here for file

Additional file 11 **Supplementary Table S9 Detailed information of top disease terms associated with non-synonymous Korean only SNVs (SNV-2/ns to SNV-10/ns)**. Click here for file

Additional file 12 **Supplementary Table S10 Shared top 10 disease terms associated with non-synonymous Korean only SNVs (SNV-2/ns to SNV-10/ns)**. Click here for file

Additional file 13 **Supplementary Table S11 Detailed information of top drug terms associated with non-synonymous Korean only SNVs (SNV-1/ns and SNV-35/ns)**. Click here for file

## Declarations

Publication costs of this article were funded by the US government.

This article has been published as part of *BMC Bioinformatics *Volume 15 Supplement 11, 2014: Proceedings of the 11th Annual MCBIOS Conference. The full contents of the supplement are available online at http://www.biomedcentral.com/bmcbioinformatics/supplements/15/S11.
