# Supplementary material for: Whole genome sequencing of 35 individuals provides insights into the genetic architecture of Korean population
Source: BMC Bioinformatics. 2014 Oct 21;15(Suppl 11):S6. doi: 10.1186/1471-2105-15-S11-S6 (PMC4251052; doi:10.1186/1471-2105-15-S11-S6)
Supplement: Additional file 1 — Supplementary Methods. [file 1471-2105-15-S11-S6-S1.docx]

**Supplementary Methods:**

**Mapping and variant calling of KPGP pipeline**

Commands:

bwa aln -I -t 3 -l 45 -k 2 ref.fa sample1_1.fq.gz > sample1_1.sai

bwa aln -I -t 3 -l 45 -k 2 ref.fa sample1_2.fq.gz > sample1_2.sai

bwa sampe -r '@RG\tID:sample\tSM:sample\tPL:Illumina' ref.fa sample1_1.sai sample1_2.sai sample1_1.fq.gz sample1_2.fq.gz > sample1.sam

samtools mpileup -ugf ref.fa sample.bam | bcftools view -bvcg - > out_file.bcf

bcftools view out_file.bcf | vcfutils varFilter -d 5 -D 150 > out_file.vcf

**Mapping and variant calling of SOAPsnp pipeline**

Commands:

soap –a sample1_1.fq.gz –b sample1_2.fq.gz –D ref.fa.index –m -x 1000 -l 44 -s 75 -v 5 -r 1 -p 8 –o sample1.PESoap -2 sample1.PESoapSingle –u sample1.PESOAPunmapped

ls sample1.PESoap sample1.PESoapSingle >soap.list

msort –k 8,n9 soap.out > soap.out.sorted

soapsnp -r 0.0005 -e 0.001 -t -u -L 90 -Q i –i soap.out.sorted –d refchr.fa –o chr.cns –M chr.cns.matrix

**Scripts for preprocessing raw genotype file of 1KGP**

#! /usr/bin/perl

use strict;

use warnings;

die "Usage: perl $0 rawdata output.dir \n" unless(@ARGV == 2);

# specify raw genotype file

my $file1 = shift;

# specify output path

my $outdir =shift;

# specify output file name

my $outname;

if ($file1 =~/(ALL.chr\S+).inte\S+vcf.gz/){

$outname="$1.geno";

}

open IN, "gunzip -c $file1|" or die $!;

open OUT, ">$outdir/$outname" or die "cannot create $!";

while (<IN>){

chomp;

next if ($_!~ /^\S+/);

next if (/^##/);

my @array = split ("\t", $_);

if (/^#CHROM/){

# generate header (format:#chr\tpos\trsID\trefalt\tsample1name\tsample2name...)

print OUT "$array[0]\t$array[1]\t$array[2]\tGENO";

foreach my $iw (9..$#array){

print OUT "\t$array[$iw]";

}

print OUT "\n";

}

else{

# select SNV lines and generate genotype for each individual

#select SNVs according to the “INFO” column

next if ($array[7]!~/VT\=SNP/);

$array[2]=~s/rs//;

my $geno ="$array[3]$array[4]";

print OUT "$array[0]\t$array[1]\t$array[2]\t$geno";

#decipher the allele meanings of the numbers used in “GT” for each individual

my %hash;

my $i=0;

$hash{$i}=$array[3];

if(length($array[4])>1){

foreach my$k (1..length($array[4])){

$hash{$i+$k}=substr($array[4],$k-1,1);

}

}else{

$hash{$i+1}=$array[4];

}

foreach my $ii (9..$#array){

if($array[$ii] =~/(\S)\|(\S)\:/){

my $left=$1;

my $right =$2;

if (defined($hash{$left})&&(defined $hash{$right})){

my $tag = "$hash{$left}$hash{$right}";

print OUT "\t$tag";

}else{

print "warning:$array[$ii]\t";

}

}

}

print OUT "\n";

}

}

close IN;

close OUT;
