# Supplementary figures and images for: Whole genome sequencing of 35 individuals provides insights into the genetic architecture of Korean population
Source: BMC Bioinformatics. 2014 Oct 21;15(Suppl 11):S6. doi: 10.1186/1471-2105-15-S11-S6 (PMC4251052; doi:10.1186/1471-2105-15-S11-S6)

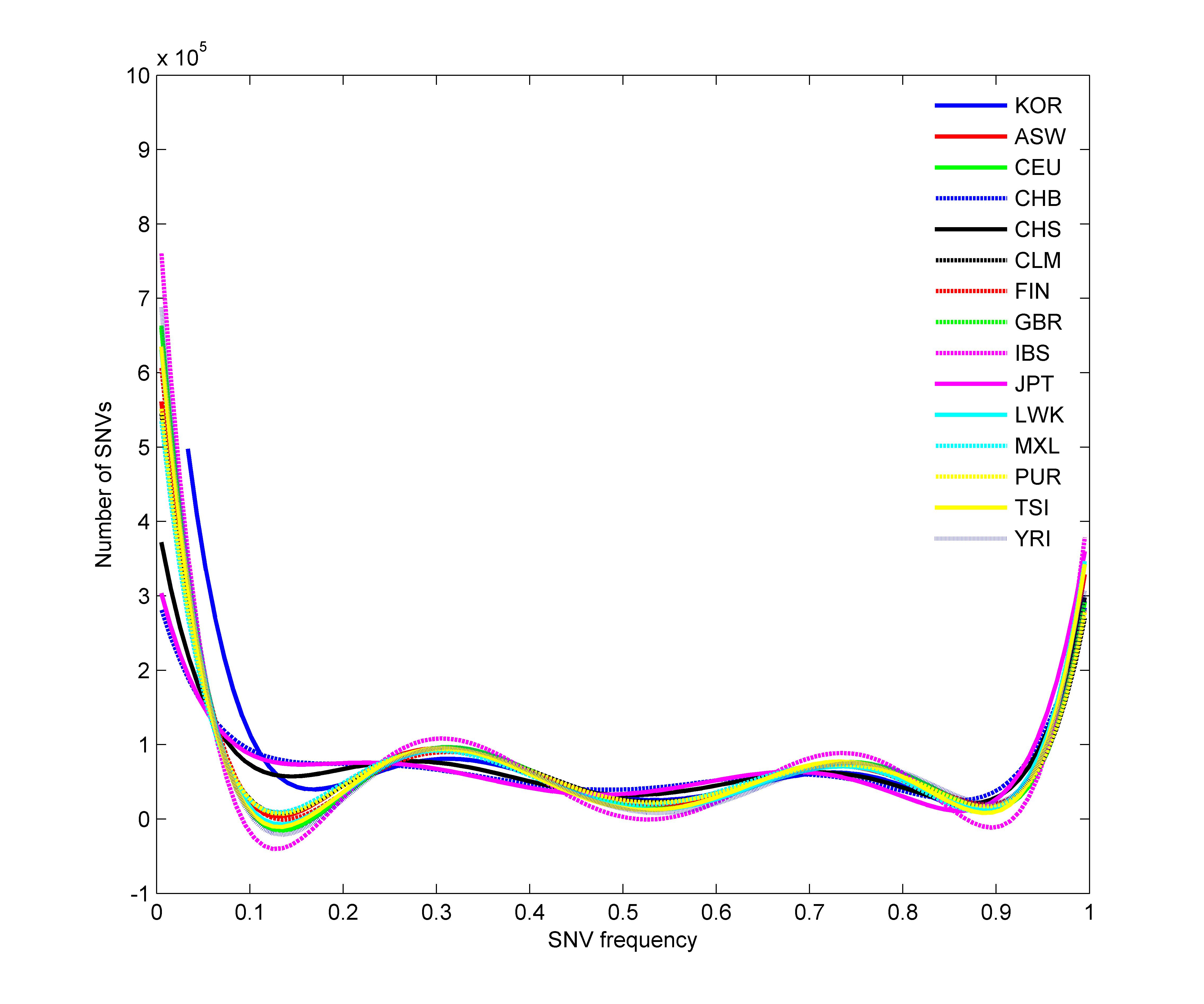

Supplement: Additional file 5 — Supplementary Figure S1 SNV frequency distributions in 15 populations. Frequency distributions in terms of occurrences in the populations were calculated for the SNVs detected in both Korean and the 14 populations from 1KGP. Populations: KOR (35 Koreans in our study), ASW (people with African ancestry in Southwest United States), CEU (Utah residents with ancestry from Northern and Western Europe), CHB (Han Chinese in Beijing, China), CHS (Han Chinese South, China), CLM (Colombians in Medellin, Colombia), FIN (Finnish in Finland), GBR (British from England and Scotland, UK), IBS (Iberian populations in Spain), JPT (Japanese in Tokyo, Japan), LWK (Luhya in Webuye, Kenya), MXL (people with Mexican ancestry in Los Angeles, California), PUR (Puerto Ricans in Puerto Rico), TSI (Toscani in Italia), and YRI (Yoruba in Ibadan, Nigeria). [file 1471-2105-15-S11-S6-S5.jpg]
